# Decoding Pedophilia: Increased Anterior Insula Response to Infant Animal Pictures

**DOI:** 10.3389/fnhum.2017.00645

**Published:** 2018-01-23

**Authors:** Jorge Ponseti, Daniel Bruhn, Julia Nolting, Hannah Gerwinn, Alexander Pohl, Aglaja Stirn, Oliver Granert, Helmut Laufs, Günther Deuschl, Stephan Wolff, Olav Jansen, Hartwig Siebner, Peer Briken, Sebastian Mohnke, Till Amelung, Jonas Kneer, Boris Schiffer, Henrik Walter, Tillmann H. C. Kruger

**Affiliations:** ^1^Institute of Sexual Medicine and Forensic Psychiatry and Psychotherapy, Kiel University, Medical School, Kiel, Germany; ^2^Department of Neurology, Kiel University, Medical School, Kiel, Germany; ^3^Department of Radiology and Neuroradiology, Kiel University, Medical School, Kiel, Germany; ^4^Danish Research Centre for Magnetic Resonance, Centre for Functional and Diagnostic Imaging and Research, Copenhagen University Hospital Hvidovre, Hvidovre, Denmark; ^5^Department of Neurology, Copenhagen University Hospital Bispebjerg, Copenhagen, Denmark; ^6^Institute for Sex Research and Forensic Psychiatry, University Medical Center Hamburg-Eppendorf, Hamburg, Germany; ^7^Department of Psychiatry and Psychotherapy, Charité – Universitätsmedizin Berlin, Berlin, Germany; ^8^Institute of Sexology and Sexual Medicine, Charité – Universitätsmedizin Berlin, Berlin, Germany; ^9^Department of Psychiatry, Social Psychiatry and Psychotherapy, Section of Clinical Psychology and Sexual Medicine, Hannover Medical School, Hannover, Germany; ^10^Division of Forensic Psychiatry, LWL-University Hospital Bochum, Bochum, Germany

**Keywords:** pedophilia, child sex abuse, baby schema, nurturing behavior, parental investment, insula, fMRI

## Abstract

Previous research found increased brain responses of men with sexual interest in children (i.e., pedophiles) not only to pictures of naked children but also to pictures of child faces. This opens the possibly that pedophilia is linked (in addition to or instead of an aberrant sexual system) to an over-active nurturing system. To test this hypothesis we exposed pedophiles and healthy controls to pictures of infant and adult animals during functional magnetic resonance imaging of the brain. By using pictures of infant animals (instead of human infants), we aimed to elicit nurturing processing without triggering sexual processing. We hypothesized that elevated brain responses to nurturing stimuli will be found – in addition to other brain areas – in the anterior insula of pedophiles because this area was repeatedly found to be activated when adults see pictures of babies. Behavioral ratings confirmed that pictures of infant or adult animals were not perceived as sexually arousing neither by the pedophilic participants nor by the heathy controls. Statistical analysis was applied to the whole brain as well as to the anterior insula as region of interest. Only in pedophiles did infants relative to adult animals increase brain activity in the anterior insula, supplementary motor cortex, and dorsolateral prefrontal areas. Within-group analysis revealed an increased brain response to infant animals in the left anterior insular cortex of the pedophilic participants. Currently, pedophilia is considered the consequence of disturbed sexual or executive brain processing, but details are far from known. The present findings raise the question whether there is also an over-responsive nurturing system in pedophilia.

## Introduction

Some men experience sexual attraction to prepubescent children or to children alt early stages of puberty. This condition is called pedophilia. While a number of accompanying neuropsychological and neurofunctional aspects of pedophilia were uncovered in recent years (Tenbergen et al., 2015) the etiology of pedophilia remains unknown.

Usually pictures of children are elicitors of nurturing behavior, particularly if the infant stimuli closely match Konrad Lorenz’s notion of baby schema (i.e., large head, large eyes, small nose, round face, and a protruding forehead) (Lorenz, 1943; Glocker et al., 2009; Borgi et al., 2014). Several functional magnetic resonance imaging (fMRI) studies, however, suggest that in pedophiles, pictures of naked children or pictures of children in swim suits trigger sexual processing (Walter et al., 2007; Sartorius et al., 2008; Schiffer et al., 2008a,b; Poeppl et al., 2011; Ponseti et al., 2012; Habermeyer et al., 2013). In a previous study, we found increased brain processing in pedophiles even in response to pictures of child faces relative to adult faces (Ponseti et al., 2014). This finding shows that the preference specific brain response of pedophiles does not depend on the presentation of infant genitals or an infant silhouette. This gives rise to the assumption that increased brain responses to infant stimuli in pedophilia are rather a consequence of an over-active nurturing system than of an over-active sexual system. Following this line of reasoning, we hypothesized that nurturing stimuli trigger increased brain responses in pedophiles even in the absence of any sexual meaning.

**FIGURE 1:**
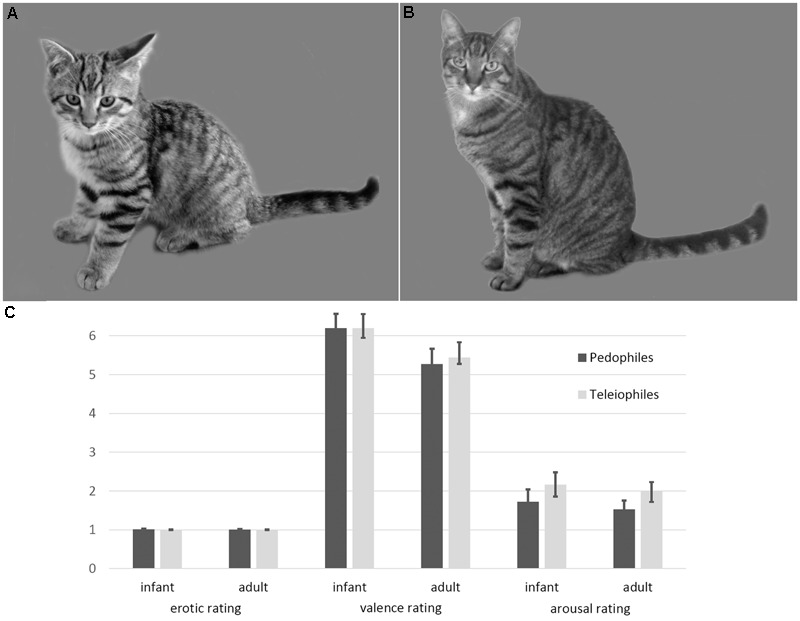
Examples and ratings of infant animal **(A)** and adult animal **(B)** stimuli. Infant animals as well as adult animals were not perceived as sexually arousing, by either pedophilic or teleiophilic raters. Similarly, valence and arousal ratings of infant animals and adult animals did not differ between pedophiles and teleiophiles **(C)**. Likert-type rating scales allowed ratings between 1 (not at all) and 9 (maximum intensity).

To test this hypothesis we exposed pedophilic and teleiophilic (i.e., those who are sexually attracted to adults) participants to pictures of adult and infant mammals (dogs, cats, rabbits, pigs, bears; **Figure 1**) during an fMRI session. We used pictures of infant and adult animals because animal infants (like human infants) are powerful elicitors of nurturing behavior, particularly if the infant stimuli closely match the baby schema (Sherman et al., 2009; Golle et al., 2013; Borgi et al., 2014). Moreover, and importantly, pictures of animal infants usually do not elicit sexual meaning or sexual arousal in pedophilic subjects in contrast to pictures depicting human children. By using infant animal pictures, we disentangled stimulus features that elicit nurturing processing from stimulus features that elicit sexual processing.

With respect to brain processing of nurturing stimuli in pedophiles, we were particularly interested in the anterior insula for the following reasons: Non-human female primates commonly raise their offspring without male cooperation (Chapais, 2008). It is supposed that paternal investment evolved with the advent of enduring pair bonding (Chapais, 2008). There is increasing evidence that about two million years ago the cognitive challenges of pair-bonding – the precondition of living in large groups in human evolution (Chapais, 2008) – induced expansion of the human brain (Dunbar and Shultz, 2007). Brain expansion is not only characterized by a general increase in neocortical volume and particularly by the increase of frontal lobe areas (Teffer and Semendeferi, 2012) but also by an expansion of the anterior part of the insula. It is suggested that the anterior insula has no equivalent in monkeys (Craig, 2009). A number of functions are associated with the anterior insula. Among these are decision-making, time perception, pain, self-recognition, empathy, and, last but not least, maternal affiliation (Craig, 2009). The anterior insula is thought to enable the most recent evolutionary step of self-awareness, which is based on primary interoceptive representations dependent on the posterior and mid-insular cortices (Craig, 2009). Therefore, the anterior insula is crucial for the emotional communication with conspecifics; it is considered to be part of a “parental caregiving neural network” (Abraham et al., 2014) and repeatedly found to be activated when adults see pictures of babies (Leibenluft et al., 2004; Noriuchi et al., 2008; Lenzi et al., 2009; Strathearn et al., 2009; Caria et al., 2012; Wittfoth-Schardt et al., 2012; Abraham et al., 2014). Persistent sexual preferences for infants are not known in non-human primates. It is therefore possible that pedophilia involves brain areas that evolved after pan-homo split. Based on our assumption that sexual interest in infants is related to over-active nurturing processing, novel brain structures that are involved in male nurturing behavior appear as likely candidates where aberrant nurturing processing take place. This applies to the anterior insula cortex. Hence, we hypothesized that nurturing stimuli receive additional processing resources in the anterior insula of pedophiles relative to teleiophiles. In addition, we supposed that increased brain responses to nurturing stimuli will not be limited to the anterior insula and will be found in other brain areas of pedophiles as well. For this reason, our analysis was both, applied to the whole brain and focused on the anterior insula as region of interest (ROI).

Currently, pedophilia is related to some kind of sexual (Cohen et al., 2002; Quinsey, 2003; Schiltz et al., 2007; Walter et al., 2007; Cantor et al., 2008; Seto, 2012) or executive malfunctioning (Schiffer et al., 2007; Eastvold et al., 2011; Krüger and Schiffer, 2011; Schiffer and Vonlaufen, 2011). However, previous findings showing some kind of mal-functioning mostly involved pedophiles that already committed child sex offending. By carefully comparing pedophiles with and without a history of child sex offenses recent studies revealed that mal-functioning is mostly restricted to those that committed child sex offenses (Kargel et al., 2016; Massau et al., 2017b; Schiffer et al., 2017). Accordingly, the neurobiological underpinnings of pedophilia remain unknown. Our hypothesis represents a novel perspective on pedophilia that is additionally motivated by the unresolved etiology of pedophilia.

## Materials and Methods

### Participants

A total of 150 male subjects were exposed to animal pictures during an fMRI session at three collaborative research sites. To make the study groups comparable as possible we matched the group members according several variables. This led to a reduction of participants per group. After group matching, we analyzed fMRI data of 115 subjects (Kiel [*n* = 67], Berlin [*n* = 33], Hannover [*n* = 15]). Sixty subjects met ICD-10 (World Health Organization, 1992) diagnostic criteria for pedophilia. Groups were matched for age (mean [SD], pedophiles: 36.6 [10.7] years, teleiophiles: 35 [10.2] years; *T*_(113)_ = 0.8; *P* = 0.42, two-sided), intelligence as estimated by means of four subtests derived from the German version of the Wechsler Adult Intelligence Scale, (WAIS-IV) (von Aster et al., 2006) (pedophiles: 41.5 [11.1] scaled sum score, teleiophiles: 43.1 [9.1] scaled sum score; *T*_(113)_ = -0.83; *P* = 0.41, two-sided), handedness as measured by a handedness inventory (Oldfield, 1971) (pedophiles: 52 right-handed, teleiophiles: 50 right-handed; Chi-square_(1)_ = 1.06; *P* = 0.37, two-sided), and sexual gender orientation (55% heterosexual pedophiles, 45% non-heterosexual pedophiles, 62% heterosexual teleiophiles, 38% non-heterosexual teleiophiles; Chi-square_(1)_ = 0.55; *P* = 0.57, two-sided). Thirty-five of the 60 pedophilic participants reported of having committed child-sex-abuse (hands-on-delict) in the past.

According to self-reports, 33 (55%) of the participants with pedophilia were sexually attracted to girls (i.e., hetero-pedophiles) and 27 (45%) to boys only, or to both boys and girls (i.e., non-hetero-pedophiles). Thirty-two (53%) of the pedophilic participants declared that they were sometimes sexually attracted to adults as well (i.e., non-exclusive pedophiles). Of the non-exclusive pedophiles 20 (63%) were heterosexual and 12 (38%) non-heterosexual. Twenty-five (42%) participants with pedophilia had previously committed sexual offenses against children. Four participants with pedophilia used psychotropic medication. The healthy control group (also referred to as teleiophiles) was recruited from the community via advertisements and included 55 men without a history of criminal behavior or current psychiatric disorders. Thirty-four control subjects (62%) were sexually attracted to females and the remaining 21 (38%) to males only, or to both males and females (i.e., non-hetero-teleiophiles). All pedophilic and teleiophilic participants declared not being sexually aroused by animals.

The pedophilic subjects were recruited either from four outpatient departments of forensic psychiatry or sexual medicine located in Berlin, Hamburg, Hannover, and Kiel, or from the community. Recruitment from the community was done by using the official NeMUP^1^-website^2^ as well as by various German Internet forums to inform self-identified pedophilic men about the study.

### Assessment

Based on a structured interview, we verified that participants had no neurological disorders, acute episodes of alcohol or drug abuse/dependence, claustrophobia, implants, or other metallic parts inside their body. We conducted a semi-structured interview to determine sexual preference with regard to age and gender, as well as the offense history of a given participant. Sexual gender and age orientation were confirmed by means of a modified version of the Kinsey scale for developmental stages (Kirk et al., 2000). In cases of uncertainty regarding the sexual age orientation, a viewing-time paradigm (Pohl et al., 2015), legal information (if available), and individual case conferences were utilized to ensure valid clinical diagnoses. The Structured Clinical Interview for the DSM (SCID) (Wittchen et al., 1997) was completed to assess for DSM-IV-TR (American Psychiatric Association, 2000) Axis I and II disorders. Global intelligence was estimated by means of four subtests (“Similarities,” “Vocabulary,” “Block Design,” and “Matrix Reasoning”) derived from the WAIS-IV (von Aster et al., 2006). We performed imaging in a second session.

This study was carried out in accordance with the recommendations of the ethics committee of the Medical School at Kiel University, the ethics committee of Hannover Medical School at University of Hannover, and the ethics committee at Medical School, Otto-von-Guericke-University Magdeburg (on behalf of the ethic committee of Charité, Universitätsmedizin Berlin) with written informed consent from all subjects. All subjects gave written informed consent in accordance with the Declaration of Helsinki. The protocol was approved by the above named ethics committees. All methods were approved by the ethics committee of the Medical School at Kiel University, the ethics committee of Hannover Medical School at University of Hannover, and the ethics committee at Medical School, Otto-von-Guericke-University Magdeburg (on behalf of the ethic committee of Charité, Universitätsmedizin Berlin), and carried out in accordance with the corresponding guidelines. Informed consent was obtained from each participant for every portion of the study in which they participated.

### Stimuli and Procedure

In a behavioral pilot experiment, thirty colored images of infant mammals (dogs, cats, rabbits, pigs, bears) and thirty colored images of adult mammals of the same species were rated by 17 pedophilic adults and 17 teleiophilic adults on a nine-point Likert-type scale in terms of sexual arousal, valence, and unspecific arousal (Bradley et al., 1993). We did not ask the subjects who participated in the fMRI measurement for sexual ratings of animals in order not to trigger sexual thoughts toward animals. Hence, raters did not participate in the fMRI measurements.

In the main experiment, we exposed participants (*n* = 150, see above) to the animal images in a block design with a total of six blocks. Three blocks consisted of ten infant animals each and another three blocks of ten adult animals each. Infant and adult animal blocks alternated. Pictures were presented for 1.5 s each and without gaps, resulting in a block length of 15 s (“task”). The inter-block interval (“rest”) was 15 s. Hence, stimulus presentation lasted for slightly less than 3 min. We instructed participants to watch the stimuli attentively. Using Presentation^®^ software (Neurobehavioral Systems) and a video projector, we projected the stimuli onto a screen behind the MRI which the participants watched via a mirror fixed on top of the head coil.

### Imaging Parameters, Preprocessing, and Statistical Analyses

All images were acquired on three separate 3 Tesla MRI scanners equipped with 32 channel head coils: a Siemens Skyra, a Siemens Trio, and a Philips Achiva. T2-weighted images on all scanner types were obtained using an echo planar imaging (EPI) sequence (slices = 38, field of view = 624 mm, voxel size = 2.3 mm × 2.3 mm × 3 mm, time of repetition = 2400 ms, echo time 30 ms, flip angle = 80°, distance factor = 10%). High-resolution T1-weighted structural images were acquired using a magnetization-prepared rapid acquisition gradient echo (MPRAGE) sequence (slices = 192, field of view = 256 mm, voxel size = 1 mm × 1 mm × 1 mm, TR = 2500 ms (Siemens)/8.4 ms (Philips), TE = 4.37 ms (Siemens)/3.9 ms (Philips), flip angle = 7°, distance factor = 50%). Axial slices were acquired parallel to the anterior-posterior commissural plane. Data preprocessing and statistical analysis were performed with SPM8 software (Wellcome Department of Cognitive Neurology, London, United Kingdom). EPI images were realigned and normalized by coregistration to the individual T1-weighted images. The non-linear spatial normalization function was determined by SPM’s segmentation function. The normalized images were spatially smoothed with a kernel of 8 mm^3^ (full width at half maximum) to ameliorate differences in intersubject localization.

For individual subject analysis (first-level), a general linear model for fMRI time series was specified using separate block regressors for each stimulus condition (infant animal and adult animal) and six regressors with movement parameters as estimated in the realignment step. Low-frequency drifts in the BOLD signal were removed by a high-pass filter with a cut-off of 128 s. Stimulus-related responses were convolved with the standard hemodynamic response function. Regression coefficients (parameter estimates) for all regressors were estimated within a subject-specific, fixed-effects model (Friston et al., 1995). Contrast images were calculated for each stimulus condition and for the differential activity between the stimulus conditions (i.e., infant animal, adult animal, infant – adult).

Group analysis was done by means of three independent second-level random effects analyses. The first second-level analysis was done by submitting the differential first-level contrast images (infant – adult animal) of the pedophilic and the teleiophilic group into a two-sample *t*-test analysis design with two additional covariates of no interest for the scanner sites. Statistical maps were computed to investigate whether the differential response to infant animals versus adult animals differs between pedophiles and teleiophiles. We set the significance threshold at *P* < 0.05 using a family-wise error (FWE) correction at the cluster level based on a primary cluster defining threshold of *P* uncorrected < 0.001 at the voxel-level (Eklund et al., 2016). Given our *a priori* interest in the anterior insula, we defined an anterior insula ROI by a sphere of 8 mm centered on the MNI coordinates *X* = -33, *Y* = 23, and *Z* = -1 for the left anterior insula ROI and *X* = 36, *Y* = 23, and *Z* = 4 for the right anterior insula ROI. We obtained these coordinates by performing a meta-analysis (Eickhoff et al., 2009) that was based on studies that reported anterior insula activation in response to human infant or animal infant stimuli (Leibenluft et al., 2004; Noriuchi et al., 2008; Lenzi et al., 2009; Strathearn et al., 2009; Caria et al., 2012; Abraham et al., 2014). The meta-analysis provided four anterior insula cluster (uncorrected for multiple comparisons) of which we used the two biggest cluster for the ROI definitions. Next we analyzed differences in brain responses to infant versus adult animals within each group of participants independently. To this end, a second-level analysis using a full factorial design, again with scanner sites as covariates of no interest, was applied to the pedophilic participant group and the teleiophilic participant group independently. First level contrast images of the infant animal stimulus condition and of the adult animal stimulus condition entered these second-level analyses. We calculated statistical difference maps of infant animal pictures relative to adult animal pictures (and vice versa). Significance threshold was set at *P* < 0.05 using a FWE correction at the cluster level based on a primary cluster defining threshold of *P* uncorrected < 0.001 at the voxel-level (Eklund et al., 2016). The resulting maps were mask (inclusive at *P* = 0.05) with the second-level maps of the corresponding condition. For instance, brain responses of the pedophiles to infant animals relative to adult animals were masked by the pedophiles’ group response to the infant animals. By doing so, we restricted the differential response map (of infant – adult) to those brain areas related to the processing of infant animals.

## Results

### Behavioral Data

Ratings of the pedophilic raters did not differ from the teleiophilic raters, with respect to sexual arousal (mean [SD] infant mammals: pedophiles 1 [0.05], teleiophiles 1 [0], *T* = 1.16 *P* = 0.25; adult mammals: pedophiles 1 [0.02], teleiophiles 1 [0.02], *T* = 0.65 *P* = 0.52), to valence (infant mammals: pedophiles 6.2 [1.5], teleiophiles 6.2 [1], *T* = 0 *P* = 1; adult mammals: pedophiles 5.3 [1.6], teleiophiles 5.4 [0.7], *T* = -0.4 *P* = 0.69), or to arousal (infant mammals: pedophiles 1.7 [1.3], teleiophiles 2.2 [1.3], *T* = -1 *P* = 0.32; adult mammals: pedophiles 1.5 [0.9], teleiophiles 2 [1.2], *T* = -1.31 *P* = 0.19; all *t*-tests were two-sided) (**Figure 1**). Pedophilic raters and teleiophilic raters did not perceive the animal pictures as sexually arousing as indicated by the corresponding mean ratings.

### Group-Differences of BOLD Responses to Animal Stimuli

**FIGURE 2:**
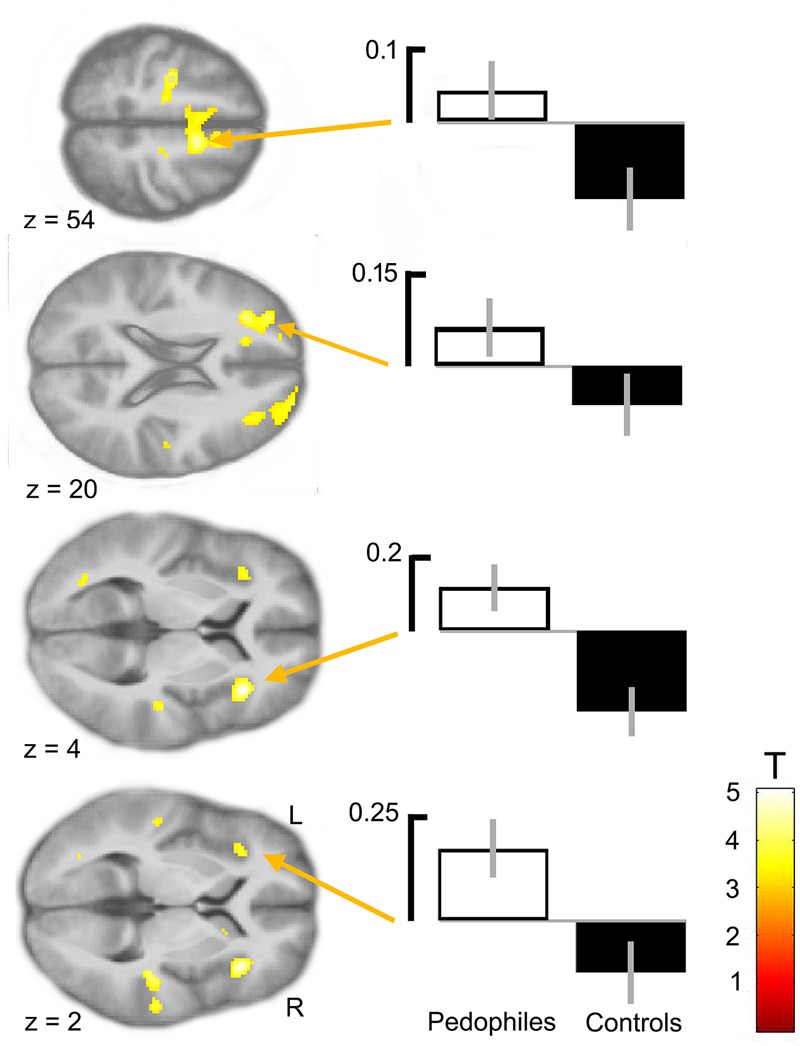
Areas of increased infant animal processing in the pedophilic group relative to the teleiophilic control group. The axial statistical *t*-score maps show elevated brain activity evoked by pictures of infant animals relative to adult animals in the pedophilic group relative to the control group (with a threshold at *P* < 0.001, uncorrected). The arrows point to the peak voxel of significant clusters as indicated in **Table 1**. The corresponding contrast estimates show differential processing of infant animals relative to adult animals in the pedophilic group and control group. The *t*-score maps were overlaid on the mean structural T1-weighted magnetic resonance image of the entire participant group. Stereotactic z coordinates of each axial slice are in MNI space.

Between-groups comparison of the differential first-level contrasts revealed an increased brain response to pictures of infant animals relative to adult animals in the pedophilic participants compared to the respective response in the teleiophilic group. We found three significant clusters: (1) The first cluster covered the right anterior portion of the insular cortex and extended to medial and superior frontal gyrus. (2) A second cluster extended from the superior frontal gyrus covering extended areas of the supplementary motor cortex, including the caudal supplementary motor area (SMA) and the rostral pre-supplementary motor area (pre-SMA) to the supplementary eye field (SEF) at both sides of the interhemispheric fissure. (3) Finally, a third cluster included the right medial frontal gyrus and superior frontal gyrus covering parts of the dorsolateral prefrontal cortex (DLPFC). Additionally, we also identified elevated brain activity in the anterior insula ROI when we applied a small volume correction (**Figure 2** and **Table 1**). In the inverse comparison (infant animals relative to adult animals in the teleiophilic participants relative to the pedophilic participants), we did not find areas of increased brain response, even when applying an uncorrected threshold of *P* < 0.001.

**Table 1:** Areas of elevated brain activity of the pedophilic participants relative to the teleiophilic participants elicited by pictures of infant animals relative to adult animals^a^.

				MNI coordinates, mm		
Brain area	Side	Cluster size, voxels	*Z*-score maxima	*x*	*y*	*z*
Pedophiles > teleiophiles (infant animals – adult animals)						
Anterior insula	R	533	4.79	36	26	4
Superior frontal gyrus	R		3.86	28	56	24
Dorsolateral prefrontal cortex	R		3.52	34	34	18
Supplementary motor area	R	473	4.27	12	2	54
Pre-supplementary area cortex	R		3.71	10	16	54
Supplementary motor area	L		3.55	-4	6	56
Dorsolateral prefrontal cortex	L	328	4.06	-28	34	20
Medial frontal gyrus	L		4.02	-28	46	16
Superior frontal gyrus	L		3.80	-18	54	16
Anterior insula^b^	L	24 (SVC)	3.59	-36	26	2

*^a^Significant clusters only FWE corrected at *P* < 0.05 on the cluster level based on a primary cluster defining threshold of *P* < 0.001 on the voxel level. ^b^Only significant with small volume correction (SVC) using a defined ROI (see section “Materials and Methods”). MNI = Montreal Neurological Institute; R = right; L = left. Cluster sizes are only given for the voxel showing peak activity.*

### Analyses of BOLD Responses within the Pedophilic and the Teleiophilic Participants

**FIGURE 3:**
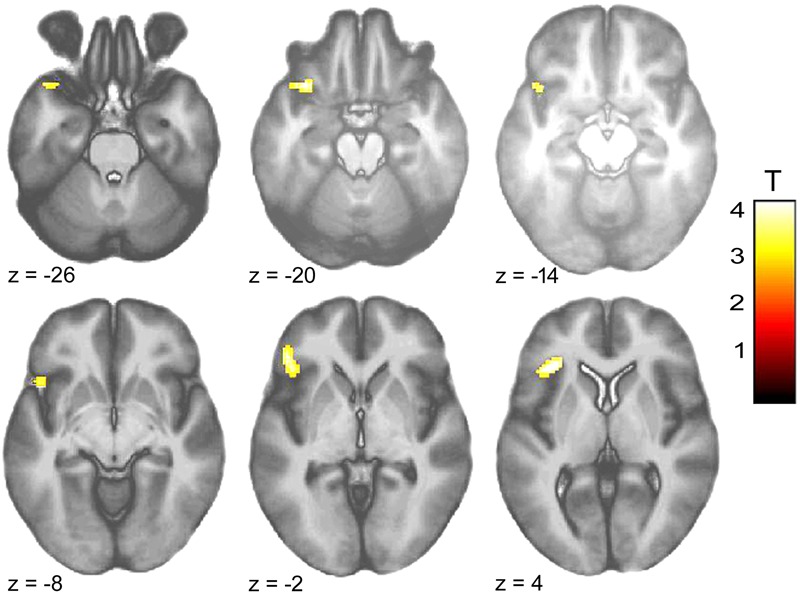
Areas of increased brain responses to infant animals in the pedophilic group. The axial statistical *t*-score maps show elevated brain activity evoked by pictures of infant animals relative to adult animals in the pedophilic group (with a threshold at *P* < 0.001, uncorrected). The cluster in the left anterior insula remains significant after applying FWE correction for multiple comparisons at *P* < 0.05 **(Table 2)**. *T*-score maps were inclusively masked by pedophiles’ *t*-score maps to infant animals. The *t*-score maps were overlaid on the mean structural T1-weighted magnetic resonance image of the entire participant group. The numbers represent the stereotactic z coordinate corresponding to each axial slice in MNI space.

Within-group comparison revealed an increased BOLD response to infant animals relative to adult animals in the pedophilic participants. When masked by the pedophiles’ brain response to infant animals, we found an area of elevated brain activity covering the left anterior insula extending ventrally along the anterior peri-insular sulcus to the adjacent frontal operculum (**Figure 3** and **Table 2**).

**Table 2:** Areas of elevated brain activity elicited by pictures of infant animals relative to adult animals in the pedophilic participants^a^.

				MNI coordinates, mm		
Brain area	Side	Cluster size, voxels	*Z*-score maxima	*x*	*y*	*z*
Pedophiles (infant animal – adult animal; masked [incl.] infant animal)						
Anterior insula	L	290	4.00	-36	26	4
Medial orbital gyrus	L		3.89	-34	18	-22
Frontal operculum	L		3.60	-46	32	0
Inferior anterior insula	L		3.54	-46	18	-10

*^a^Significant clusters only FWE corrected at *P* < 0.05 on the cluster level based on a primary cluster defining threshold of *P* < 0.001 on the voxel level. MNI = Montreal Neurological Institute; L = left. Cluster sizes are only given for the voxel showing peak activity.*

We did not detect any BOLD signal increases in response to pictures of adult animals relative to infant animals in the pedophilic participants. Within-group analysis of the teleiophilic participant’s’ brain responses did not reveal any significant signal differences between the infant animal and adult animal condition.

## Discussion

This study aimed to evaluate whether there are hints of an aberrant nurturing processing in pedophilic men. In order to study nurturing processing independent from sexual processing we exposed our pedophilic and teleiophilic subjects during an fMRI-session to pictures of infant and adult animals because these images were not perceived as sexually arousing. We found an increased neural response to pictures of infant animals in pedophiles. Increased activation was found in a network of brain regions that contribute to motivating behaviors such as nurturing, including the anterior insula (Leibenluft et al., 2004; Noriuchi et al., 2008; Lenzi et al., 2009; Strathearn et al., 2009; Caria et al., 2012; Wittfoth-Schardt et al., 2012; Abraham et al., 2014), mesial motor (Caria et al., 2012; Laurent and Ablow, 2013), and prefrontal cortex (Strathearn et al., 2009).

As hypothesized, we found the anterior insular cortex to be more engaged in pedophiles than in teleiophiles during infant animal processing. Within-group analysis revealed that the left anterior insula was particularly related to infant animal processing in pedophiles. The left anterior insula is of particular interest because previous studies with healthy adults found this area activated during the processing of infant *humans* (Leibenluft et al., 2004; Caria et al., 2012; Abraham et al., 2014). The preferred activation of the left anterior insula is in accordance with the notion of a functional lateralization of the anterior insula, which has been observed in a number of conditions including appetitive or group-oriented, affiliative emotions (Bartels and Zeki, 2004; Johnstone et al., 2006; Koelsch et al., 2006; Jabbi et al., 2007; Ortigue et al., 2007; Takahashi et al., 2008). Right anterior insula activity connoted aversive or individual-oriented (survival) emotions (Craig, 2009; Duerden et al., 2013).

Human infants [particularly if matching the baby schema (Lorenz, 1943; Glocker et al., 2009)] compared to human adults trigger increased brain responses in healthy teleiophiles (Luo et al., 2015). On a subjective level, infant animals elicit emotional, motivational, and behavioral responses that are similar to the responses elicited by human infants (Golle et al., 2013; Lehmann et al., 2013). However, previous research at the level of brain responses did not find increased responses to infant animals relative to adult animals in teleiophiles (Caria et al., 2012). For this reason, in this previous study it was argued that conscious responses to infant pets do not extend to implicit processing or – stated differently – that implicit processing of infant stimuli is species specific in order to facilitate parental nurturing behavior. Our study confirms previous findings in that infant animals do not trigger increased brain responses in teleiophiles. In addition, our findings suggest that the species-specific boundary is absent in pedophiles’ brain response to infant animals. But the differential response of pedophiles to infant animals appears to be limited to implicit processing given that the stimuli were rated equally by pedophilic and teleiophilic raters in terms of valence and arousal and the absence of sexual meaning.

Taken together, the pedophilic subjects of the present study showed an over-responding to infant animal stimuli in a network of brain regions that contribute to motivating behaviors. This is in accordance with our hypothesis that nurturing stimuli receive additional processing resources in pedophiles. It is of interest that some of the areas of increased response to infant animals are related to the mating domain. The left anterior insula, being a crucial area of nurturing processing, was also frequently found to be activated in sexual brain studies (Stoleru et al., 2012). Furthermore, the left anterior insula (as well as the SMA) is a constituent of the human attachment system, thereby enabling both nurturing and pair-bonding (Feldman, 2016). Based on both observations, (i) the over-responding to nurturing stimuli in various motivational areas and (ii) the functional overlap of nurturing and sexual processing of the involved left anterior insula a tentative and simple model of pedophilia could be as follows: Nurturing stimuli receive additional processing resources by mating-circuits. In case of human infant stimuli this leads to a sexual connotation of infant stimuli. This idea is supported by the suggestion that nurturing and pair-bonding are two closely inter-related domains in humans at the level of physiological functions, brain processing, and involved neuropeptides (Feldman, 2016). Interestingly, more than two decades ago, the Austrian ethologist Eibl-Eibesfeldt expressed a similar view suggesting that pedophilia might in some cases be based on an “eroticization of parental love” (Eibl-Eibesfeldt, 1990). According to this view, the functional division between the domains of nurturing and mating behavior in pedophilia is incomplete.

### Limitations of the Study and Recommendations for Future Research

Some alternative explanations of the present finding have to be discussed: The pedophilic response to infant animals could be the result of conditioning in the sense of repeated sexual-child stimulus pairing. However, this would require some kind of conscious sexual attraction to the conditioned (animal) stimulus, which is not the case. Furthermore, a similar effect should then be expected to occur in the teleiophilic participants with respect to adult animals, which is also not the case. Another possibility would be that pedophilic men experience an elevated emotional congruence with children and in consequence also with any type of infant stimuli. However, there were no differences in the valence and arousal ratings of the infant animals between pedophilic and teleiophilic raters.

Although it is difficult to explain the present findings by alternative explanations, the tentative model of pedophilic brain processing as delineated above is far from being proven by this experiment. First, because the functional overlap of the left anterior insula of nurturing and sexual processing does not necessarily imply that both domains are simultaneously involved in the processing of infant animal stimuli. Many brain areas fulfill different functions. Second, because our findings do not show that brain responses of pedophiles to *human* infants are driven by an over-responding nurturing system. Here we found an increased response of pedophiles to *non-human* infant stimuli and we are not able to generalize these findings to the processing of human infants. This is the consequence of our effort to disentangle sexual from nurturing processing. Albeit our study does not allow for direct inferences about the processing of human infant stimuli in pedophiles, our findings are at least compatible with the hypothesis of an increased nurturing processing in pedophiles. May be future research will be able to more accurately delineate the input of nurturing and mating networks to the processing of human infants stimuli in the brains of pedophiles. Third, the assumption of an over-responding nurturing system is solely based on the observed hemodynamic response differences to nurturing stimuli between the pedophilic and the teleiophilic subjects. The absence of behavioral correlates (in the sense of an increased fondness of pedophiles to infant pets) might be caused by a limitation to implicit processing of pedophiles’ over-responding to infant animals. However, in the absence of behavioral correlates our inference about an over-active nurturing system is mainly based on reverse inference (Poldrack, 2006). We cannot rule out the possibility that, for instance, differences in moral reasoning between the participant groups (Massau et al., 2017a) were associated with response differences to nurturing stimuli. A considerable portion of the pedophilic participants were recruited from the community or received treatment voluntarily in the out-patient departments. Possibly, these persons have particular abilities to understand the moral disvalue of sexual behavior toward children and respond therefore differently to nurturing stimuli. Under these circumstances it might be recommendable to improve the external validity of the present findings by applying another measurement of brain activity to the present paradigm, for instance by measuring evoked brain potentials. Previous studies showed that evoked brain potentials are sensitive to the baby schema effect (see for reference: Luo et al., 2015). Moreover, the measurement of pedophiles’ and teleiophiles’ evoked brain potentials in response to infant animal stimuli would allow to study the time course of group differences in more detail. Unless such an external validation isn’t done, the present findings should be considered as preliminary.

### An Anthropological Account

Virtually all female mammals exhibit nurturing whereas male nurturing is very rare (Woodroffe and Vincent, 1994). In primates, male nurturing is only known in some but not all, distantly related, monogamous species (including homo sapiens) (Fernandez-Duque et al., 2009). This indicates that paternal (in contrast to maternal) nurturing is triggered by a particular set of sexual circumstances. In some primate species, paternal nurturing was interpreted as an adaptation to the high cost of maternity (Achenbach and Snowdon, 2002; Miller et al., 2006; Ziegler et al., 2006). However, in case of humans, paternal nurturing was also interpreted as an opportunistic mating strategy (Buss, 2008; Chapais, 2008; Fernandez-Duque et al., 2009). The latter is supported by the fact that there is a great variance in human male nurturing, both within and between human societies (Fernandez-Duque et al., 2009). In any case, from an evolutionary perspective, paternal nurturing is an unusual and probably more recently evolved male behavior. Phylogenetically speaking, male brains are not “geared” to handle infant stimuli. This may explain why the “parental caregiving” network of males and females displays differences (Abraham et al., 2014).

It might be hypothesized that, in contrast to ancient maternal infant stimulus processing, human male brains process infant stimuli with brain areas that might not be associated primarily with caregiving. If so, it is conceivable that the involvement of brain areas not “geared” to the paternal nurturing aspects associated with infant stimuli makes male brains vulnerable to not maintaining the functional division between the domains of nurturing and mating behavior. This could particularly apply to those males who are affected with neurodevelopmental problems or irregularities. It could be, therefore, that the incidence of head injuries and left-handedness was found to be elevated in pedophiles (Blanchard et al., 2003, 2007). However, this should be not generalized to cases of acquired pedophilic interest and behavior after serve brain lesions (Burns and Swerdlow, 2003; Sartori et al., 2016) as there might be other factors involved as well.

May be that the current experiment opens an avenue of new approaches to research in pedophilia. For instance, it could be tested whether pedophiles’ brain responses to infant animals are related to neurodevelopmental aberrances. Next, given that human females’ response to infant faces is modulated by female sex hormones (Sprengelmeyer et al., 2009) and pituitary hormones (Strathearn et al., 2009; Gregory et al., 2015), it appears possible that pedophiles’ responses to infant features are mediated by these agents as well. In this case, new options for the pharmacological treatment of pedophilic child sex offenders would arise.

## Author Contributions

JP, AS, and BS were responsible for study concept and design. HG, AP, SM, TA, and JK were responsible for data collection. JP, DB, HL, and JN analyzed the data and wrote the manuscript. OG, SW, GD, and OJ provided neuroimaging analysis support. TK, PB, HW, and HS provided domain expertise and guidance through all stages of research and manuscript preparation. All authors revised the manuscript critically and approved the final manuscript version.

## Conflict of Interest Statement

The authors declare that the research was conducted in the absence of any commercial or financial relationships that could be construed as a potential conflict of interest.

## Footnotes

1 “NeMUP” is the acronym of a German research consortium funded by the German Federal Ministry for Education and Research and stands for Neural Mechanisms Underlying Pedophilia and Sexual Offending Against Children.

2 www.nemup.de
